# Flow-enhanced priming of hESCs through H2B acetylation and chromatin decondensation

**DOI:** 10.1186/s13287-019-1454-z

**Published:** 2019-11-27

**Authors:** Jiawen Wang, Yi Wu, Xiao Zhang, Fan Zhang, Dongyuan Lü, Bing Shangguan, Yuxin Gao, Mian Long

**Affiliations:** 1 Center for Biomechanics and Bioengineering, Key Laboratory of Microgravity (National Microgravity Laboratory) and Beijing Key Laboratory of Engineered Construction and Mechanobiology, Institute of Mechanics, Chinese Academy of Sciences, Beijing, 100190 China; 2 School of Engineering Science, University of Chinese Academy of Sciences, Beijing, China

**Keywords:** Embryonic stem cell, Fluid shear mechanomics, Nuclear spreading, Histone acetylation, Chromatin decondensation

## Abstract

**Background:**

Distinct mechanical stimuli are known to manipulate the behaviors of embryonic stem cells (ESCs). Fundamental rationale of how ESCs respond to mechanical forces and the potential biological effects remain elusive. Here we conducted the mechanobiological study for hESCs upon mechanomics analysis to unravel typical mechanosensitive processes on hESC-specific fluid shear.

**Methods:**

hESC line H1 was subjected to systematically varied shear flow, and mechanosensitive proteins were obtained by mass spectrometry (MS) analysis. Then, function enrichment analysis was performed to identify the enriched gene sets. Under a steady shear flow of 1.1 Pa for 24 h, protein expressions were further detected using western blotting (WB), quantitative real-time PCR (qPCR), and immunofluorescence (IF) staining. Meanwhile, the cells were treated with 200 nM trichostatin (TSA) for 1 h as positive control to test chromatin decondensation. Actin, DNA, and RNA were then visualized with TRITC-labeled phalloidin, Hoechst 33342, and SYTO® RNASelect™ green fluorescent cell stain (Life Technologies), respectively. In addition, cell stiffness was determined with atomic force microscopy (AFM) and annexin V-PE was used to determine the apoptosis with a flow cytometer (FCM).

**Results:**

Typical mechanosensitive proteins were unraveled upon mechanomics analysis under fluid shear related to hESCs in vivo. Functional analyses revealed significant alterations in histone acetylation, nuclear size, and cytoskeleton for hESC under shear flow. Shear flow was able to induce H2B acetylation and nuclear spreading by CFL2/F-actin cytoskeletal reorganization. The resulting chromatin decondensation and a larger nucleus readily accommodate signaling molecules and transcription factors.

**Conclusions:**

Shear flow regulated chromatin dynamics in hESCs via cytoskeleton and nucleus alterations and consolidated their primed state.

## Background

Embryonic stem cells (ESCs) derived from blastocysts of early embryos have the capability of self-renewal and pluripotency to form any cell types [1], serving as a perfect cell model in organism development, drug discovery, and regenerative medicine [2]. Under physiological conditions, stem cells reside within a local microenvironment consisting of biomechanical and biochemical cues that are essential for their stemness and direct their differentiation to specific cell types. A thorough understanding of these regulatory factors is required to manipulate stem cell behaviors [3]. While most of the recent studies focus on biochemical signaling pathways, increasing evidence indicates that mechanical factors also contribute to these processes [4].

Cells experience various mechanical stimuli such as fluid shear, contractile force, and mechanical stretch during development and morphogenesis. For example, various physiological flows (e.g., cilia- and vascular-driven flows or low-amplitude interstitial flow) are generated in living tissues [5], which manipulate tissue morphogenesis at distinct developmental stages. In early mammalian embryos, the dynein-driven cilia create a leftward nodal flow at the node by rotating in one specific direction and the signaling molecules induced by the flow determine specification of the left-right axis [6]. The embryonic heart tube begins to form and elongate on the right as the first visible structure of left-right asymmetry at late stage [7], in which the fluid flow generated by the heart tube reshapes the blood islands formed by embryonic mesodermal cells into mature vessels [8]. These observations suggest that mechanical stimuli are indispensable for ESC stemness, differentiation, and organogenesis.

Inspired by these physiologically relevant mechanical processes, in vitro mechanical control of behaviors of embryonic stem cells also attracts much attention. For instance, shear stress promotes the differentiation from ESCs to hematopoietic cells through upregulation of *RUNX1* [9]. Shear stress contributes to ESC differentiation to vascular wall cells by activating the underlying signaling pathways [10, 11]. Since in these studies, mechanical manipulation in embryonic stem cell differentiation is usually coupled with biochemical factors such as differentiation-inducing factors, it is still elusive on the role of shear flow in ESC differentiation.

ESCs originated from distinct organism present different features or states. Mouse ESCs (mESCs) could be classified into a naive and a primed pluripotent states in vitro, where the naive mESCs exhibit a grounder state pluripotency while the primed mESCs show a limited pluripotency and are primed for lineage specification [12, 13]. Many biological features differ significantly between these two states, such as colony morphology, growth factor requirement for maintaining undifferentiated growth, gene expression profile, and *X* chromosome inactivation [14]. Alternatively, human ESCs (hESCs) are shown to be at the primed state [15]. Meanwhile, accumulating evidence shows that ESC nuclei at different pluripotent states have distinctive stiffness and auxeticity, partly driven by the alterations of epigenetic modification and chromatin state. In this regard, nuclear mechanics could manipulate gene expression through transcriptional factors and molecular turnover in the nucleus during ESC differentiation [16, 17]. Although recent studies indicate that the external forces and geometric constraints can regulate nuclear morphology and the nuclear volume can modify chromatin organization [18–22], the potential interplay among mechanical stimuli, nuclear morphology, nuclear mechanoepigenetics, chromatin organization, gene expression regulation, and pluripotent states is still an open issue in ESC differentiation.

Upon the *mechanomics* concept proposed recently to define all the possible mechanical stimuli the cells experience and the global molecular responses the cells make [23], we carried out the first mechanomics study on hESCs in vitro under shear flow, attempting to reveal the mechanical responses and their physiological significances in hESC fate decision. With functional tests in a typical case of steady laminar flow, we found that shear forces were able to be transmitted to the nuclei of hESCs via CFL2/F-actin cytoskeleton and thus translated into biochemical signaling through H2B acetylation to regulate chromatin organization, suggesting that the nucleus could serve as the major mechanosensor and play a key role in mechanical control of hESC priming, at least, under fluid shear.

## Materials and methods

### Reagents

Rabbit polyclonal antibodies were used against CFL2 (Abcam, ab96678, 1:50 for western blotting (WB) and 1:100 for immunofluorescence (IF) staining), acetyl-histone H2B (Lys12) (Cell Signaling Technology, #5410, 1:100 for WB), and histone-H2B (acetyl) (Abcam, ab1759, 1:200 for IF), respectively. Rabbit monoclonal antibodies (mAbs) were used against β-actin (Cell Signaling Technology, #12620, 1:200 for WB), histone-H2B (Cell Signaling Technology, #12364, 1:100 for WB), LMNB1 (Abcam, ab133741, 1:50 for WB), POU5F1 (Cell signaling, #5177, 1:50 for IF), and NANOG (Abcam, ab195018, 1:100 for IF), respectively. Mouse mAb was used against histone-H2B (Alexa Fluor® 488 conjugate; Abcam, ab204258, 1:500 for IF). Anti-rabbit Detection Module was purchased from ProteinSimple (#DM-001) for WB. DyLight® 594-conjugated donkey anti-rabbit second antibodies were from Abcam (1:200 for IF). Actin was visualized with phalloidin-TRITC-labeled mixed isomers (Sigma, P1951, 5 μg/mL for IF). DNA was visualized with Hoechst 33342 (Life Technologies, H3750, 1:500 for IF).

### Cell culture and treatment

hESC line H1 was cultured on matrigel-coated polystyrene surface in DMEM/F12 basal medium supplemented with mTeSR1 (Stem Cell Technologies, Vancouver, BC, Canada). Cells were cultured at 37 °C and humidified 5% (v/v) CO_2_ atmosphere with medium exchange every 24 h. For passage, cells were digested with dispase (Stem Cell Technologies, Vancouver, BC, Canada) and collected by centrifuging at 1000 rpm × 10 min after harvesting with mechanical scraping. Cells were then resuspended gently and dispensed into new culture plates. In some cases, cells were treated with 200 nM trichostatin A (TSA) for 1 h as positive control.

### Application of fluid shear

**Fig. 1:**
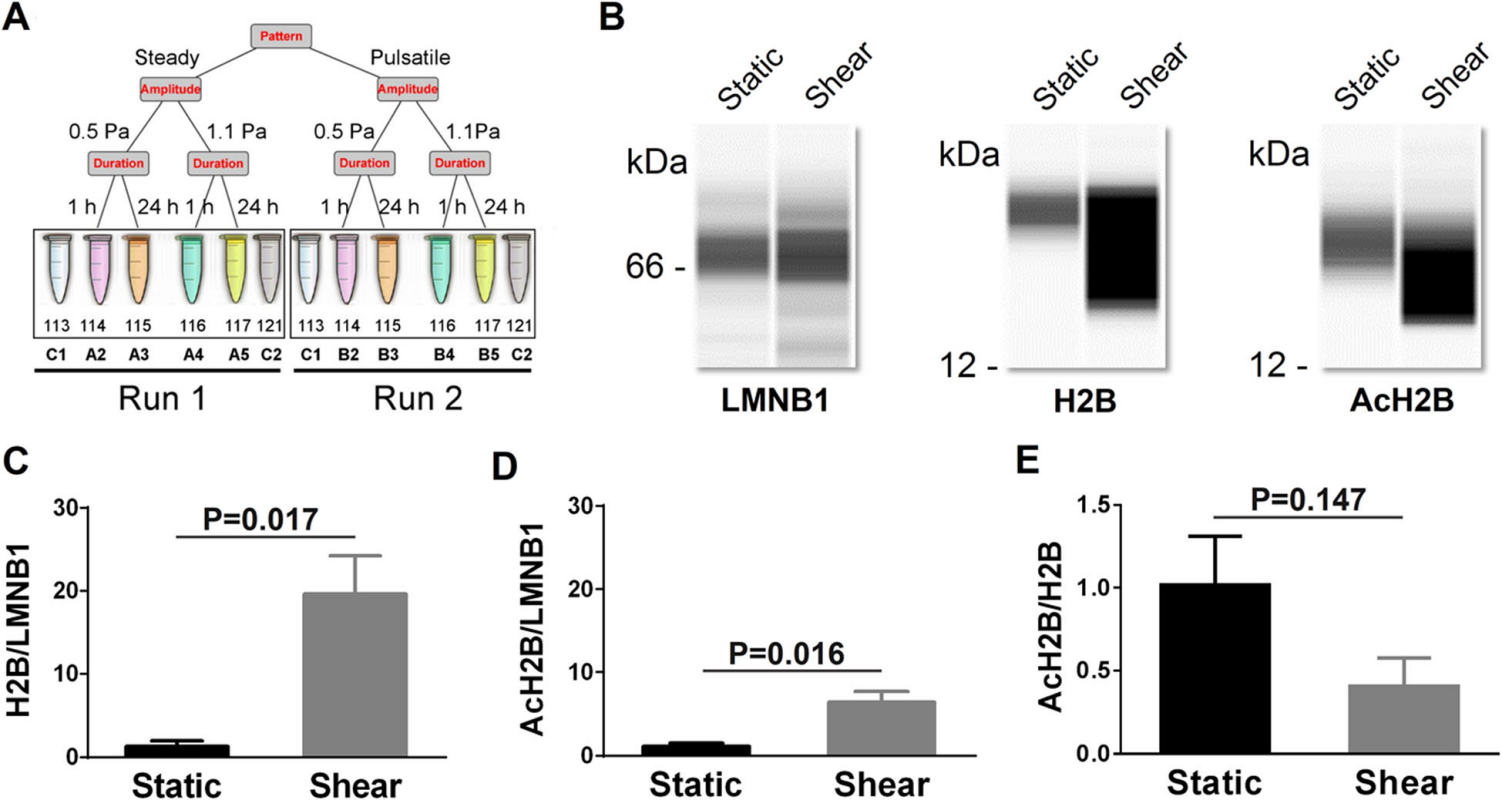
Shear flow induces histone H2B acetylation of hESCs. The schematic of iTRAQ labeling (**a**) was illustrated, where two samples (C1, C2) were used as static control in both LC-MS/MS Run 1 and Run 2. Simple western immunoblots (**b**) showed H2B and AcH2B proteins under static and shear conditions. Expression abundance of H2B (**c**) and AcH2B (**d**) as well as the ratio of AcH2B/H2B (**e**) were validated with immunoblots. LMNB1 served as loading control in **b**–**e**. Data are presented as the mean ± SE of normalized chemiluminescence in three replicates. Here Shear in **b**–**e** denotes the steady shear flow of 1.1 Pa for 24 h

The cells after digestion were seeded onto the substrate of a parallel-plate flow chamber pre-coated with matrigel. The seeded cells were incubated for 48 h prior to exposure to shear flow. Then, shear stress was applied using a customized flow chamber unit (Additional file 1: Figure S1) at given mechanical types, patterns, and parameters, which are summarized in Fig. 1a. Cultured cells were kept at 37 °C and humidified 5% (v/v) CO_2_ atmosphere when being sheared and then harvested or fixed immediately after the mechanical stimulation at given duration indicated. Two to three replicates for the cells exposed to fluid shear were conducted, and all the proteins collected were pooled together for quantification using isobaric tag for relative and absolute quantitation (iTRAQ).

### Mass spectrometry (MS) analysis

Proteins collected in each case were reduced, alkylated, and quantified using Bradford method. One hundred micrograms of protein was digested and then labeled with six-channel iTRAQ reagent (Applied Biosystems, 4390812) upon the protocol given in the kit. Labeled samples were pooled and fractionated by strong cation exchange (SCX) chromatography (Phenomenex Luna SCX, 250 × 4.60 mm, 100 Å) on an HPLC system (Agilent 1100). All peptide samples collected were purified using an online Nano-LC system (Dionex ultimate 3000 nano LC system) and analyzed by a Q-Exactive hybrid quadrupole-orbitrap MS (Thermo Fisher). MS/MS data were filtered using PD software (Proteome Discover 1.3, Thermo Fisher) and then searched with Mascot (version 2.3.0, Matrix Science) against NCBI_human database. Protein abundance was quantified using PD. iTRAQ ratio for each protein was defined as geometric mean of the ratio compared to technical duplicate of control samples.

### Simple western analysis

Nuclear and cytoplasmic extracts were isolated with a Nuclear and Cytoplasmic Protein Extraction Kit (Bioteke, Beijing, China) upon the manufacturer’s instructions. Both the extracts were quantified using a BCA assay kit (Pierce®, Thermo Scientific, USA). Simple western analysis was performed using the WES™ device (ProteinSimple, San Jose, CA, USA) upon the manufacturer’s protocols. In brief, 3 μL of proteins was loaded on the plate, labeled with a biotin reagent, and detected by chemiluminescence using streptavidin-horseradish peroxidase. The proportion of the protein of interest was then measured for its nuclear or cytoplasmic fraction, in comparison to the biotinylated ladder. Data were analyzed using Compass™ software (Version 4.0.0, ProteinSimple). Each protein peak was measured automatically and the median area under the peak was normalized to the LMNB1 for nuclear protein or the ACTB for cytoplasmic protein.

### Immunofluorescence (IF) microscopy

The cell samples were fixed in either pre-chilled methanol at − 20 °C for 10 min for RNA staining or 4% (w/v) formaldehyde (Solarbio) for 15 min and permeabilized with 0.4% (w/v) Triton X-100 (Solarbio) for 15 min for protein detection. To visualize RNAs, samples were stained with SYTO® RNASelect™ green fluorescent cell stain (Life Technologies). To visualize target proteins, samples were blocked with 1% (w/v) BSA (Bovogen) for 1 h at 37 °C, incubated with primary antibodies or TRITC-labeled phalloidin for actin overnight at 4 °C, washed with PBS, and then incubated with appropriate fluorescent-labeled secondary antibodies for 1 h at 37 °C. After washing in PBS, appropriate amount of Hoechst 33342 was added to stain nuclei for 10 min at RT. The samples were washed with PBS and then mounted in antifade reagent (Life Technologies) overnight. Finally, the stained cells were imaged at RT using confocal laser scanning microscopy (Zeiss L710, Germany).

### Determination of cell stiffness

Cells were cultured in static control or under fluid shear for a given duration, prior to a mechanical test by atomic force microscopy (AFM) (BioScope Catalyst, Bruker, Germany) at RT. Eight positions were tested in each colony using a standard pyramidal tip, silicon nitride cantilever with a spring constant of 0.0106 N/m. All the indentations were performed at the same scanning rate of 1.00 μm/s. Force-displacement curves at a given point were repeated ten times and fitted individually using a Sneddon model to get Young’s modulus [24]. Mann-Whitney’s test was used to determine statistical significance between static control and corresponding flow samples. *p* < 0.05 was set as the statistical significance threshold.

### Quantitative real-time PCR (qPCR) analysis

The expressions of two typical stemness biomarker genes *POU5F1* and *NANOG* were determined via qPCR analysis. After lysing the cells, RNAs were extracted using RNAprep Pure Micro Kit (TIANGEN, Beijing, China) upon the manufacturer’s instructions. cDNAs were synthesized using oligo primers and a reverse transcription kit (TOYOBO, Japan). qPCR analysis was performed using QuantStudio 7 Flex (Thermo Fisher Scientific). All gene expression levels were normalized to the housekeeping gene *GAPDH*. The following primers were used:

**Table:** 

Name	Forward	Reverse
*GAPDH*	ACCACAGTCCATGCCATCA	TCCACCACCCTGTTGCTGTA
*NANOG*	AGCCTCTACTCTTCCTACCACC	TCCAAAGCAGCCTCCAAGTC
*POU5F1*	GACAACAATGAAAATCTTCAGGAGA	TTCTGGCGCCGGTTACAGAACCA

### Cell apoptosis assays

The cells were harvested, and Annexin V-PE apoptosis detection kit I (BD Biosciences) was used to quantitatively determine the percentage of cells that are actively undergoing apoptosis. A flow cytometer (FACSCanto, BD Biosciences) was used to collect the data, and FlowJo software (version 7.6.1, BD Biosciences) was used to analyze the results.

### Image analysis

Confocal or optical images were analyzed using ImageJ (version 1.50i, National Institutes of Health). ImageJ built-in measure tools were used to determine cell density and area (*Analyze Particles*) or protein colocalization (*Colocalization*) on the basis of intensity correlation analysis (ICQ) [25]. Both the pseudopodium and periphery of single hESC colonies were outlined manually, and the pseudopodium number and perimeter were then measured. To estimate the cytoskeletal protein expressions, the minimal fluorescence intensity within a colony was subtracted as mean background for CFL2/F-actin staining while the mean intensity outside the regions of the interest (ROIs) was subtracted as mean background for DNA, H2B, or AcH2B staining. The significances of data consistent with normality distribution, after checking normality using Shapiro-Wilk’s normality test, were assessed using two-tailed unpaired Student’s *t* test, while the others that do not satisfy normality assumption were assessed using Mann-Whitney’s test. All the significances for multiple comparisons were corrected using the false discovery rate (FDR) method. *p* < 0.05 was set as the statistical significance threshold.

### Gene set enrichment analysis

For each protein, the three-way ANOVA test was performed to get *p* value and fold change (FC) corresponding to each mechanical parameter (pattern, amplitude, and duration of shear flow). Protein was then ranked by its FC value minus unity to represent the up- and downregulated expression with resulted positive and negative values, respectively. Gene set enrichment analysis (GSEA) was conducted using javaGSEA Desktop Application (version 3.0). The pre-ranked protein list and msigdb.v6.2.symbols.gmt gene set were used for running the tool “GSEAPreranked” with default parameters.

## Results

### Mechanomics analysis of hESCs

To identify mechanosensitive proteins under fluid shear, hESC line H1 was subjected to physiologically significant shear flow (Fig. 1a) with a customized flow chamber (Additional file 1: Figure S1). Steady and pulsatile flow patterns were defined upon in vivo mechanical microenvironment with respective parameters of amplitude, frequency, or duration. Here the shear stress was set to be 0.5 and 1.1 Pa [9, 26], 1- and 24-h durations were given to present short- and long-term effect [27], and the frequency of pulsatile flow was fixed at 1 Hz [28], all of which were physiologically related parameters known in the literatures. Protein samples collected from this mechanically induced culture were labeled in parallel with four iTRAQ labels. Other two labels were used as controls in each run, and two runs corresponding to the two distinct flow patterns were analyzed by MS (Fig. 1a).

To test the biological effects of shear stress, gene set enrichment analysis (GSEA) was used and a varying degree of enrichment of “protein acylation,” “protein acetylation,” “ribosome,” “nuclear matrix,” “nuclear periphery,” and “positive regulation of cell adhesion” gene sets was found (Additional file 2: Figure S2). Except the “positive regulation of cell adhesion,” all other terms were related to nucleus or epigenetics, implying that the nucleus may serve as the mechanosensing element for hESCs under various shear flows and then induces epigenetic alternations or mechanoepigenetic responses [29].

### Flow-induced alterations in histone acetylation and chromatin state

To address the above hypothesis, the functions of typical mechanosensitive proteins were tested for hESCs subjected to fluid shear. Hereafter a typical case of steady flow with 1.1 Pa for 24 h was used for extensive functional tests to exemplify the impact of shear flow on hESC behaviors. The pattern of steady flow was typically chosen to mimic physiological flow in early embryo development before heart formation [8], the stress amplitude of 1.1 Pa was to represent its critical role in controlling differentiation [30], and the loading duration of 24 h was to investigate the long-term [31] and adaptive alterations [32] of hESCs. Specifically, histone H2B was picked up as the candidate from all the mechanosensitive proteins related to acetylation under shear flow, not only because it is one of the most duration-sensitive proteins (*p* value = 0.01, FC = 1.30) but also a structural protein that interacts with DNA and induces chromatin organization via acetylation, methylation, phosphorylation, ubiquitination, and sumoylation [33]. Here a simple WB test was performed to further validate the differential expression of nuclear H2B and acetylated H2B (AcH2B) (Fig. 1b). The results showed that both H2B and AcH2B were upregulated (Fig. 1c, d) under fluid shear, even though the ratio of AcH2B/H2B was comparable between static control and shear flow (Fig. 1e).

**Fig. 2:**
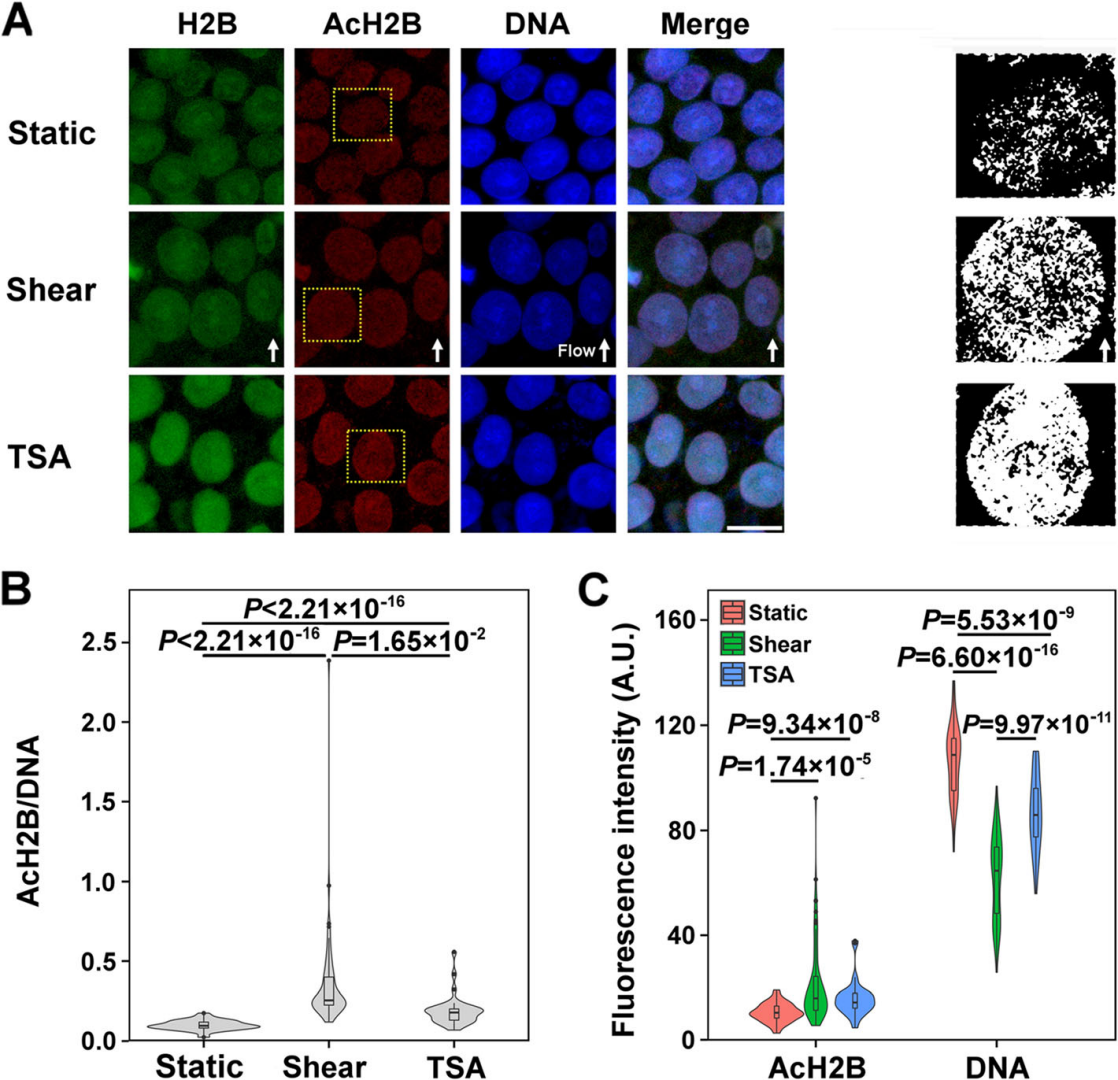
Acetylation of histone H2B and chromatin state under fluid shear. Histone acetylation was shown in (**a**) with typical immunofluorescent images of H2B, AcH2B, and DNA (left three columns) as well as the threshold transformation of AcH2B (right column; corresponding to the respective yellow boxes in the 2nd column from the left). Also plotted were the measured fluorescence intensities corresponding to AcH2B/DNA ratio (**b**) and the mean AcH2B and DNA intensities (**c**). The numbers of tested replicates and measured colonies, (M, m), are (4, 49) for control, (3, 65) for fluid shear, and (3, 34) for TSA treatment. Flow direction was indicated by solid arrows in **a**. Bar = 20 μm in **a**. Here Shear denotes the steady shear flow of 1.1 Pa for 24 h

We further tested the H2B acetylation using a typical APKK^12^(Ac)GSK^15^(Ac) KAVYC acetylation site of H2B and the chromatin condensation when hESCs were presented under static control, fluid shear, or trichostatin A (TSA) treatment (positive control), a chemical inhibitor of histone deacetylase (HDAC) that increases histone acetylation. The threshold transformation of AcH2B fluorescence images showed an evident upregulation and a homogenous distribution under fluid shear or TSA treatment, identifying the decondensation of chromatin in the two cases (Fig. 2a). Noting that histone AcH2B and DNA are perfectly colocalized in nucleosomes [34], AcH2B intensity normalized to that of DNA (i.e., AcH2B/DNA) was found to be 3.5-fold enhanced under fluid shear than static control (Fig. 2b). When the cells were treated with TSA, the AcH2B/DNA level was 1.9-fold increased than static control but still lower than fluid shear (Fig. 2b), presumably due to distinct mechanisms between shear- and TSA-induced histone acetylation. To further isolate either effect of histone acetylation or chromatin condensation on these high AcH2B/DNA levels, AcH2B and DNA intensities were further analyzed respectively. Significant enhancement in AcH2B intensity and dramatic reduction in DNA intensity were observed under fluid shear or TSA treatment, as compared to those measured from static control (Fig. 2c). DNA intensity was reduced more significantly under fluid shear than TSA treatment even at similar AcH2B intensities in the two cases, suggesting that fluid shear presents a stronger effect on chromatin decondensation than TSA treatment and induces predominantly the high AcH2B/DNA level.

### Flow-induced changes in nuclear morphology and mechanics

**Fig. 3:**
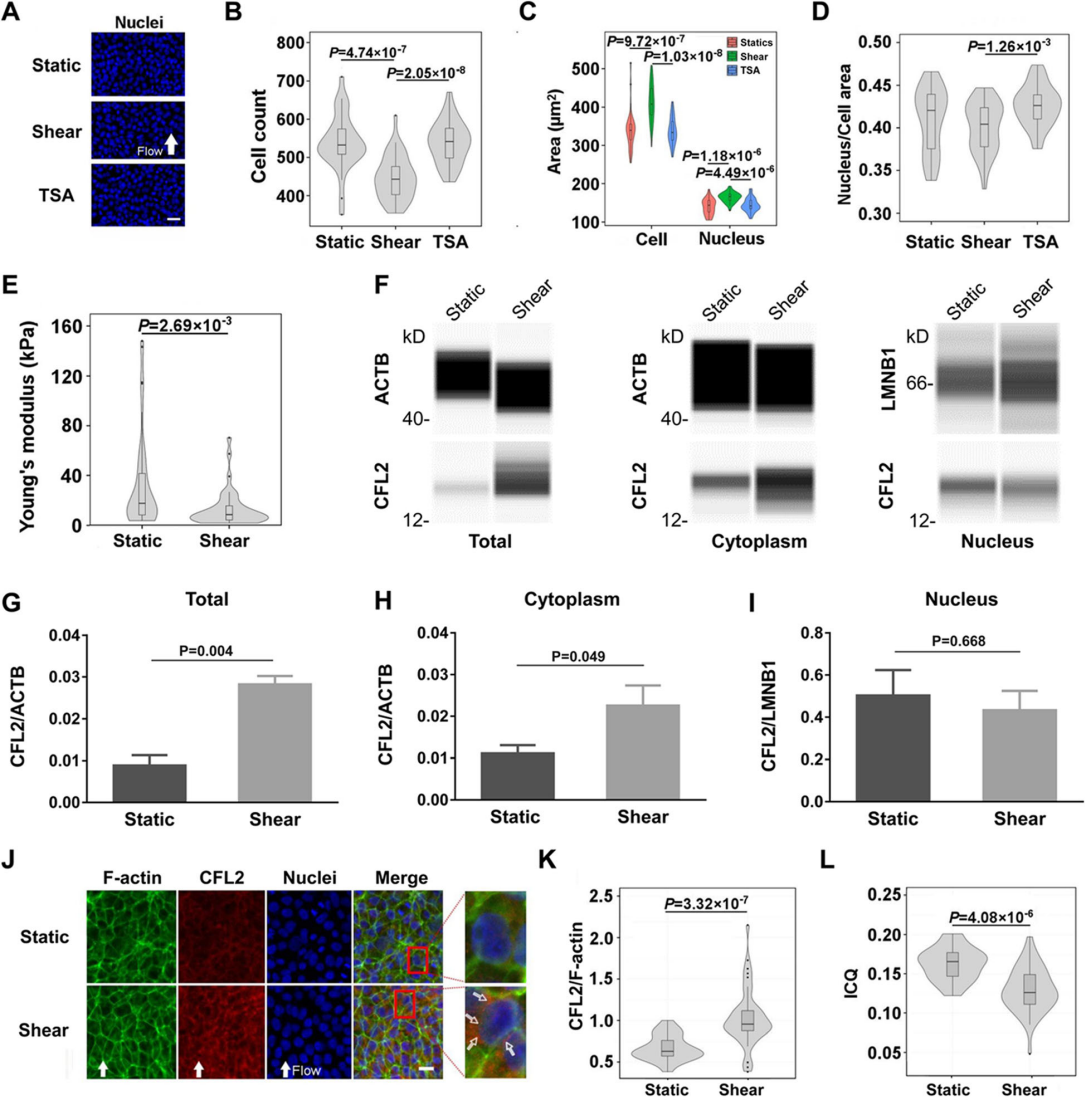
Nucleus spreading and cytoplasmic CFL2/F-actin reorganization under steady shear flow of 1.1 Pa for 24 h. DNA was visualized with Hoechst 33342 (**a**) for quantifying cell density (**b**), cell or nucleus area (**c**), as well as nucleus/cell area ratio (**d**). Nucleus stiffness was measured using AFM assay and presented by their Young’s moduli (**e**). Simple western immunoblots (**f**) showed CFL2 proteins extracted from the whole cell, cytoplasm, or nucleus under static and shear conditions. Here ACTB served as the loading control for total and cytoplasmic proteins and LMNB1 as the loading control for nuclear proteins. CFL2 expressions in whole cells (**g**), cytoplasm (**h**), and nucleus (**i**) were validated with immunoblots. Data are presented as the mean ± SE of normalized chemiluminescence in three replicates. Cytoskeletons (**j**) were imaged to quantify CFL2 to F-actin ratio (**k**) and intensity correlation quantile (ICQ) of F-actin and CFL2 (*L*). Open arrows in **j** indicated CFL2 aggregation close to nuclear periphery. The numbers of measured colonies and positions in each colony (m, n) are (5, 8) in **e**. The numbers of tested replicates and measured colonies, (M, m), are (4, 32) for static control, (3, 36) for fluid shear, and (3, 30) for TSA treatment in **a**–**d**, as well as (3, 24) for static control and (3, 45) for fluid shear in **k** and **l**. Flow direction was indicated by solid arrows at the bottom. Bar = 50 μm in **a** and 20 μm in **j**

Chromatin is a major component of the nucleus, and chromatin state could be regulated by nuclear shape [20]. Since fluid shear presented strong effects on chromatin decondensation, we further tested, via Hoechst staining, if this flow-induced excess chromatin decondensation results from the changes in nuclear morphology (Fig. 3a). Cell density (number per frame) was reduced by fluid shear compared to static control or TSA treatment (Fig. 3b). By contrast, cellular and nuclear areas were all increased synergistically under fluid shear (Fig. 3c), presenting a similar ratio of nucleus/cell area to static control (Fig. 3d). These results suggested that cell spreading and nuclear enlargement are sensitive to fluid shear. Interestingly, these features only happened under fluid shear rather than TSA treatment, implying the existence of distinct mechanisms between mechanical and biochemical stimuli.

Additionally, the correlation among AcH2B/DNA ratio, AcH2B or DNA expression, and nuclear spreading under fluid shear was tested using pairwise plots. It was found that AcH2B/DNA had little correlation with DNA density (*r* = − 0.0572) and nuclear area (*r* = − 0.136) under shear flow, supporting that the variation of AcH2B/DNA was regulated only by AcH2B level in this case. As expected, there existed a strong positive correlation between AcH2B/DNA ratio and AcH2B level (*r* = 0.883). A weak positive correlation was also observed between AcH2B and DNA levels (*r* = 0.331), both of which were correlated negatively with nuclear area (*r* = − 0.372 and − 0.565 respectively) (Additional file 3: Figure S3). Setting |*r*| > 0.5 as significant correlation, this analysis suggested that the nuclear enlargement is presumably responsible for chromatin decondensation while the level of AcH2B should be regulated by other factors.

It is known that nucleus morphology and chromatin state regulate nucleus mechanics [20]. Thus, the elastic modulus of the nucleus was measured accordingly by applying AFM cantilever on the top of those individual hESC cells inside a colony. This indirect protocol could be reasonable in estimating mechanical features of the nuclei, since the nucleus volume contributes the majority of cell volume in hESCs (Fig. 3d) and cell stiffness mainly depends on its nuclei [35, 36]. Collected results indicated that nucleus modulus of hESCs was significantly decreased to 13 kPa after the application of fluid shear, significantly lower than control case (33 kPa) (Fig. 3e). It was also notable that the deviation of the modulus was narrowly distributed, suggesting that mechanical properties of individual nucleus were likely uniform under fluid shear. These findings implicated a reverse correlation between nuclear stiffness and fluid shear via decondensing chromatin structure and enhancing cell spreading.

Nucleus mechanics is also associated with cytoskeletal network and nuclear matrices in a way so-called mechanical homeostasis [37, 38]. Here the proteins involved in nuclear matrix and nuclear periphery were further identified by mechanomics analysis (cf. Additional file 2: Figure S2d and e), among which CFL2, an F-actin binding protein that disassembles actin filaments, is a unique mechanosensitive protein of cytoskeletal network (Additional file 4: Table S1). To determine whether CFL2 expression and translocation mediates morphological change of the nucleus under fluid shear, CFL2 expression was first validated using the WB test (Fig. 3f). The results showed that fluid shear promoted the expression of CFL2 (Fig. 3g), or more specifically, the cytosolic CFL2 (Fig. 3h) rather than nuclear CFL2 (Fig. 3i). Both F-actin and CFL2 molecules were further co-stained to test cytoskeletal alteration (Fig. 3j). For the expression of these two molecules, the mean fluorescence intensity of F-actin was reduced under shear when the total F-actin amount remained the same due to its enlarged area (Additional file 5: Figure S4). The ratio of CFL2/F-actin varied from 0.5 in control to 1.02 under shear flow (Fig. 3k), implying that F-actin was impaired due to the disassembling effects of CFL2 on F-actin filaments. For the intracellular localization of the two molecules, the colocalized fraction of F-actin and CFL2 was suppressed from 0.163 to 0.130 (Fig. 3l). Those redundant CFL2 proteins were redistributed at the nucleus periphery (Fig. 3j, open white arrows). All these observations proposed an active cytoskeleton reorganization under fluid shear.

Taken together, fluid shear promoted the nuclear spreading of hESCs by initiating chromatin decondensation, enlarging nuclear shape, softening nucleus elasticity, and enhancing the expression of total and cytoplasmic CFL2. Cytoskeletal reorganization could transmit the shear forces to the nucleus, establishing the mechanical homeostasis under shear flow.

### Flow-enhanced H2B acetylation via cytoskeleton reorganization

**Fig. 4:**
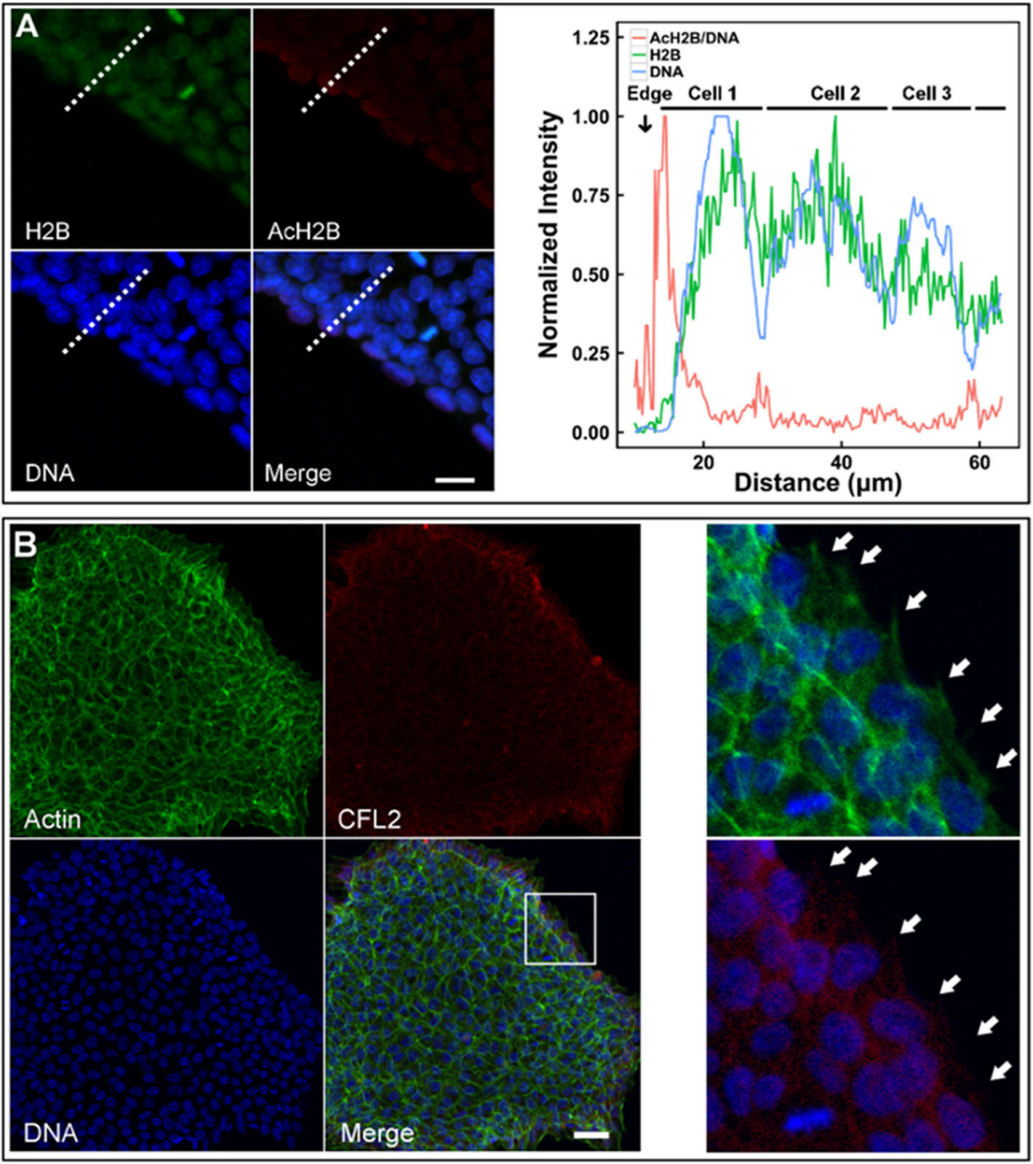
H2B acetylation and CFL2/F-actin cytoskeleton at the edge of a colony. Expressions of AcH2B/DNA, DNA, and AcH2B were quantified (**a**; red, blue, and green lines in right panel denotes, respectively, AcH2B/DNA, DNA, and H2B expressions). Also plotted were the distributed F-actin and CFL2 proteins at the edge of a typical colony (**b**). All intensity values were normalized to (0, 1) upon the formulation of <*X*_*i*_ > = (*X*_*i*_ − *X*_min_)/(*X*_max_ − *X*_min_), where *X*_*i*_ = data point *i* and *X*_min_ or *X*_max_ = the minimum or maximum value among all the data points. Solid arrows in **b** indicated the pseudopodia. Bar = 20 μm in **a** and 50 μm in **b**

Shear flow not only enhanced H2B acetylation (a biochemical event), but also altered nuclear features via cytoskeleton reorganization (a biomechanical event). Evidently, the potential cross-talking could exist between these biochemical and biomechanical cues. Here we first tested H2B acetylation in hESC colonies. Normalized intensity of AcH2B/DNA ratio increased sharply from the outer edge of the colony to reach the peak within the outmost layer, followed by quick reduction to a plateau prior to the adjacent layer along radial direction (red line). By contrast, both DNA (blue line) and H2B (green line) expressions fluctuated periodically with a similar pattern along radial direction and no peaks were found at the edge (Fig. 4a). Especially, the outmost layer of a colony provided a cell contact-free region at the periphery and presented a large amount of pseudopodia, while the cells inside the colony were surrounded by other cells without visible pseudopodia. Next, we tested the correlation of H2B acetylation at the edge of colony with the local F-actin filaments, since cofilin/F-actin dynamics generates the forces for pseudopodia extension and contraction [39, 40] and the forces are transmitted via cytoskeletons physically linked to the nucleus [41]. Distinct patterns of expression or colocalization of F-actin and CFL2 were presented between the edge and inner regions of the colony. At the edge, F-actin and CFL2 proteins were highly expressed and the nucleus was encapsulated by the fine, long, and outward F-actin filaments that are colocalized well with CFL2. Besides, F-actin and CFL2 proteins were fully spread out to form numerous pseudopodia. At the interior, nuclei were encapsulated by thick F-actin stress fiber with reduced colocalization with CFL2 (Fig. 4b). Thus, all the cues implied the positive correlation between H2B acetylation and cytoskeleton reorganization in a cell layer-specific manner.

**Fig. 5:**
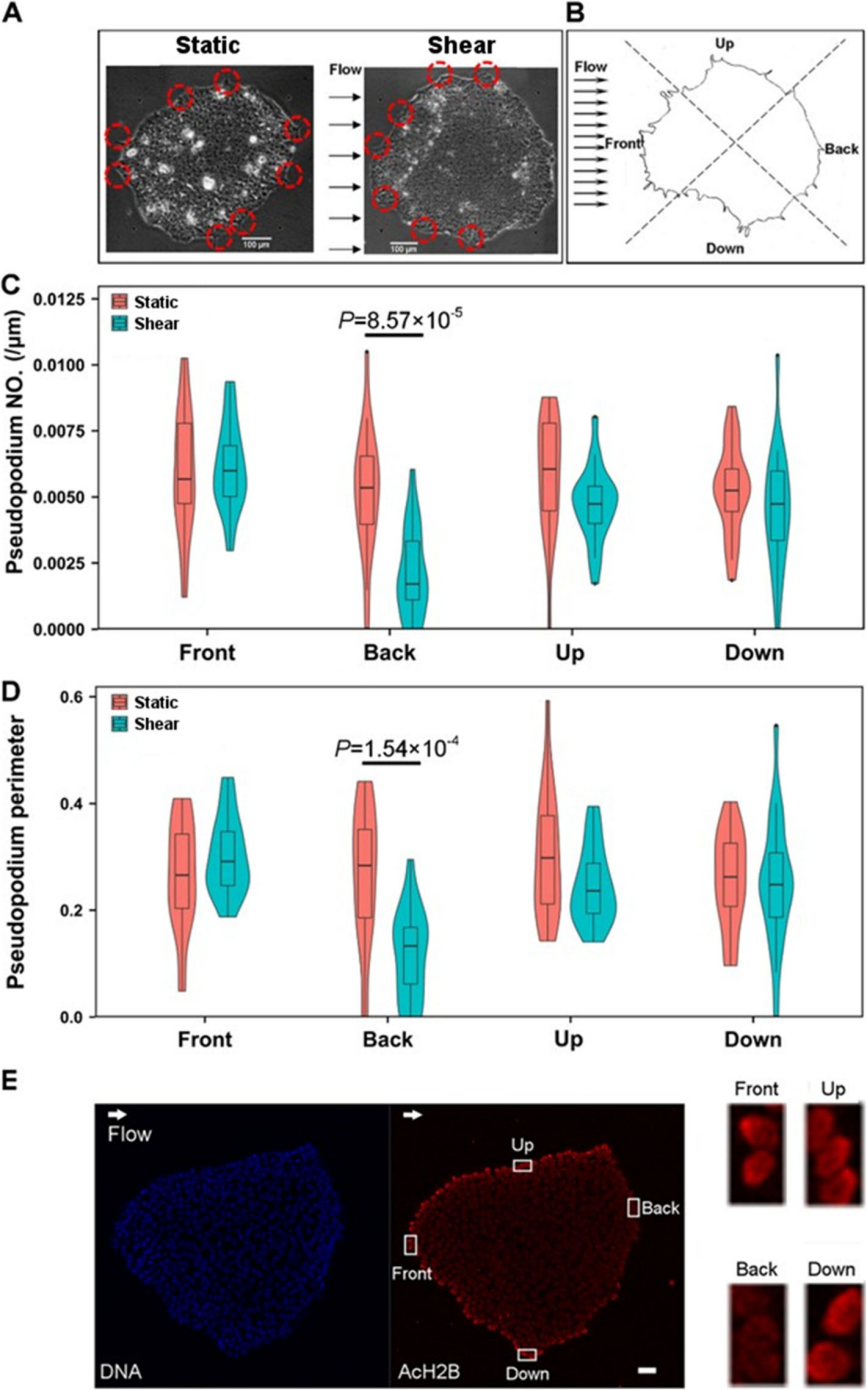
Colony polarity and H2B acetylation. Microscopic images of a typical colony were illustrated under static control or fluid shear (**a**), and the relevant analysis protocol was given for pseudopodium number and perimeter (**b**). Also plotted were the pseudopodia number per unit contour length (**c**) and total pseudopodia perimeter normalized by colony contour length (**d**) under static control or fluid shear, as well as the localized expression of H2B acetylation at the edge under fluid shear (**e**). Pseudopodia were shown in the red circles in **a**. Five colonies were measured under each condition. Bar = 100 μm in **a** and 50 μm in **e**. Here Shear denotes the steady shear of 1.1 Pa for 24 h

Another interesting feature, namely, time-dependent colony polarity, was found under shear flow when those colonies were forced to establish a retracting tail back against flow (Fig. 5a), which could be assumed as the accumulative behavior of single cell polarity [42]. By segregating a colony into four quadrants with 90° each (Fig. 5b), the pseudopodia distribution analysis displayed unique reduction of the number (Fig. 5c) and perimeter (Fig. 5d) for the pseudopodia at the back, but not for those at the front, up or down quadrant, indicating the direction-sensitive retraction of back F-actin network. This feature provided an effective way to further reveal the causality between F-actin network and H2B acetylation. Intriguingly, the cells at the back yielded lower AcH2B expression than those along other directions under shear flow (Fig. 5e). Since F-actin network in pseudopodia is dominated by cofilin/F-actin interaction [43], it was reasonably speculated that H2B acetylation results from F-actin reorganization. Given that TSA treatment could not induced the nuclear spreading (cf. Fig. 3c) and F-actin interacts with nuclear matrix and chromatin through LINC complex [44, 45], the shear forces were believed to be transmitted through F-actin network to enlarge the nucleus and further enhance H2B acetylation.

### Flow modified primed state of hESCs

**Fig. 6:**
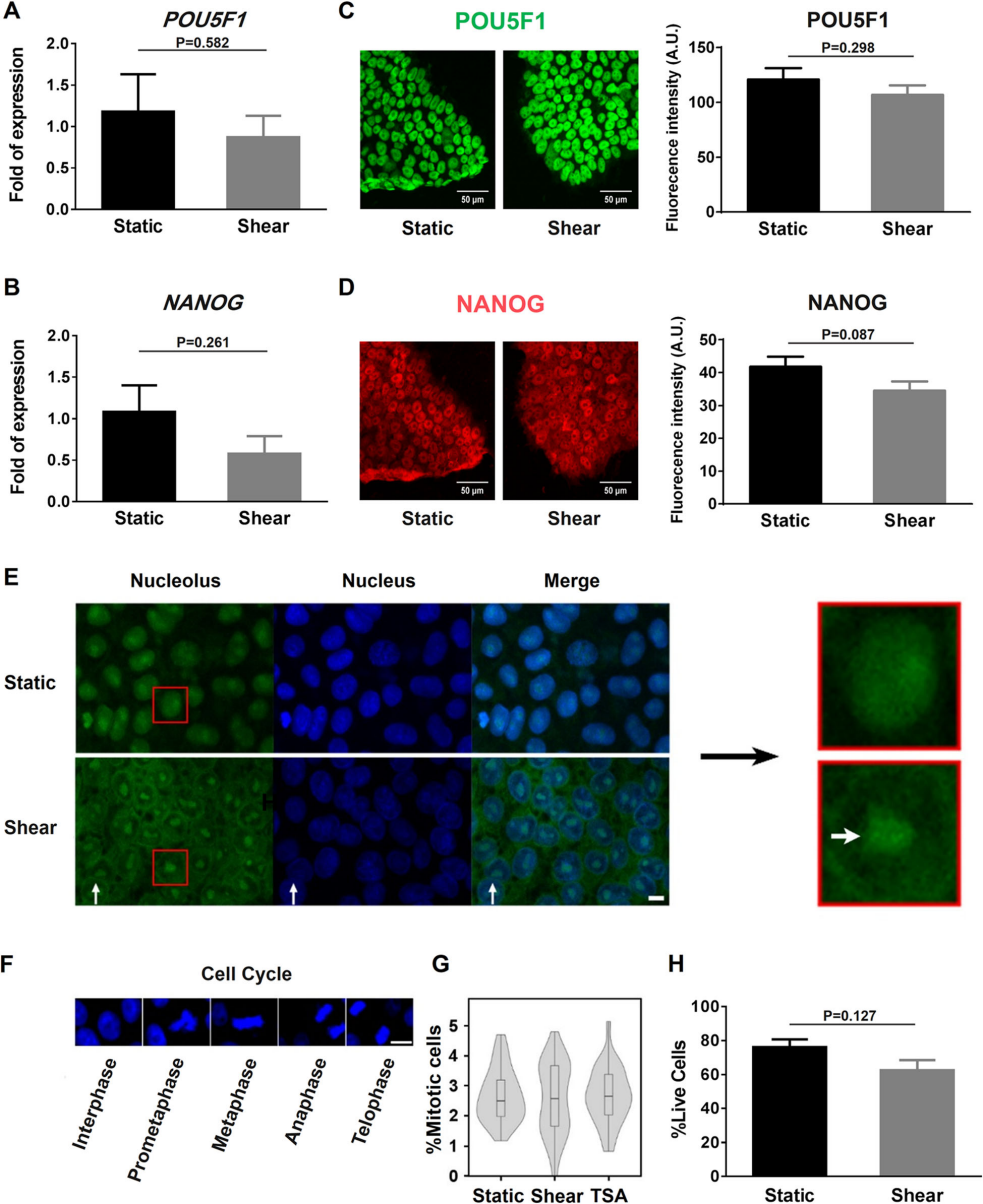
Role of fluid shear on hESC priming, cell cycle, and apoptosis. **a**, **b** *POU5F1* (**a**) and *NANOG* (**b**) expressions obtained from qPCR analysis. Data were normalized to *GAPDH* and presented as the mean ± SE in three replicates under each condition. **c**, **d** POU5F1 (**c**) and NANOG (**d**) immunostaining for hESCs under static and fluid shear conditions. Data in right columns were presented as the mean ± SE obtained from three replicates for total 60 colonies under each condition. **e** Representative images of nucleolus (indicated by thick white arrow in the most right column) for a typical colony. **f**, **g** DNA at different mitotic phases was visualized with Hoechst 33342 (blue) (**f**) to estimate the percentage of mitotic cells (**g**). Also plotted was the apoptotic percentage of hESCs using FACS analysis (**h**). The numbers of test replicates and measured colonies, (M, m), are (4, 32) for static control, (3, 36) for fluid shear, and (3, 30) for TSA treatment in **g**. Bar = 10 μm in **e** and 20 μm in **f**. Here Shear denotes the steady shear of 1.1 Pa for 24 h

hESCs cultured statically in mTeSR1 medium is considered to be at the primed state [46], which is characterized by decondensed chromatin and softened nuclei [16] and primed to differentiate. The alterations in chromatin state and nucleus mechanics observed from the current study implied that hESCs under fluid shear are still at the primed state, which is consolidated by the enhanced acetylation level and colony growth [47] as well as the existing stemness marker NANOG (Additional file 6: Figure S5). To specify the pluripotent state of hESCs under fluid shear, canonical stemness markers were tested using various techniques. Both *POU5F1* (Fig. 6a) and *NANOG* (Fig. 6b) gene expressions under shear flow were at a similar level to static control. The same pattern was also found at the protein level via POU5F1 and NANOG immunostaining tests (Fig. 6c, d). These results indicated that hESC pluripotent state is not altered by shear flow.

The state of hESCs could also be associated with the enrichment of ribosome (cf. Additional file 2: Figure S2c). Ribosomal RNAs (rRNAs) are the major structural component of ribosome, and the ribosome biogenesis happens in nucleoli [48]. Under shear flow, nucleolar-associated chromatin consisting of intra- and peri-nucleolar chromatin was visibly reduced with chromatin decondensation. By contrast, the nucleolus became much clear with confined contour and large size (Fig. 6e), indicating that more rRNAs were synthesized in the presence of shear flow. As rRNAs are necessary for maintaining ESC pluripotency [49, 50], these findings indicated that fluid shear enhances pluripotency through activating rRNA synthesis.

Functionally, this state would not affect cell cycle and apoptosis. Cell cycle phases (Fig. 6f) were first observed and no significant differences were found among static control, fluid shear, and TSA treatment, with a comparable proportion of mitotic cells (~ 2.5%) in all cases (Fig. 6g). hESCs under fluid shear also presented similar apoptosis percentages to static condition (Fig. 6h). The comparable cell cycle phases and apoptosis fractions indicated that hESCs under shear flow still kept their self-renewal capacity.

Collectively, these observations indicated that hESCs under fluid shear still maintain their stemness and self-renewal and are apart away from differentiating into lineage-specific cells. However, the primed state was consolidated by fluid shear, which could be characterized by more decondensed chromatin, more softened nuclei, and high nuclear spreading.

## Discussion

**Fig. 7:**
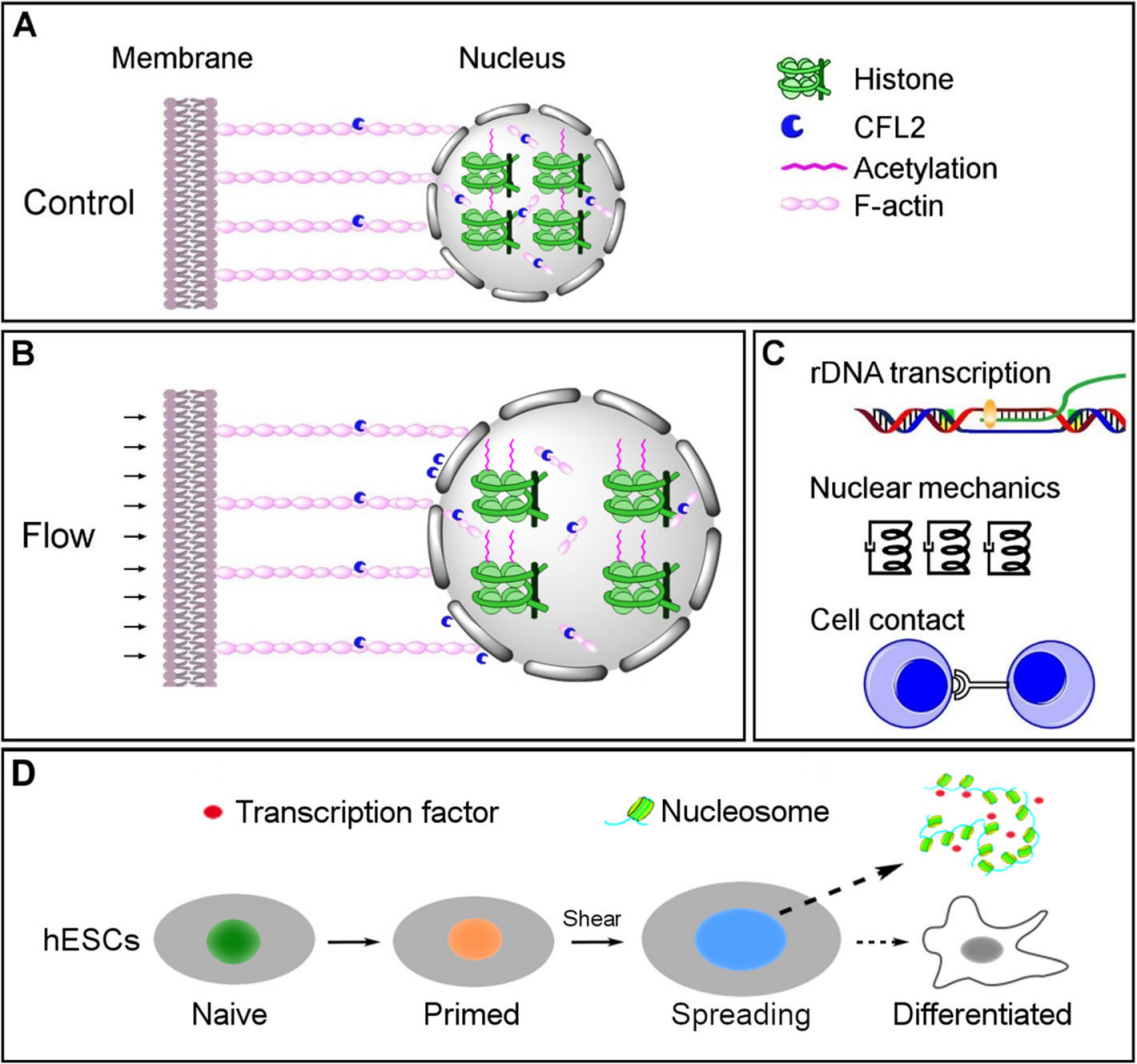
Working model of flow-induced hESC priming. H2B acetylation level is low under static control (**a**). Fluid shear increases the nuclear area via CFL2/F-actin cytoskeletal remodeling (biomechanical signaling) and then enhances the chromatin decondensation via H2B acetylation (biochemical signaling) (**b**). In succession, chromatin decondensation enforces the nucleolus formation but softens the nucleus, while H2B acetylation is correlated negatively with cell-cell contact (**c**). Consequently, chromatin accessibility to transcription factors is enhanced in hESCs to consolidate their primed state (**d**)

Stem cells live in a complex and diverse physiological microenvironment to maintain tissue homeostasis [23, 51, 52]. Since the first report on matrix elasticity-directed stem cell differentiation [53], a large number of studies have been reported on mechanical control of lineage specification of stem cells [11]. To elucidate how shear stress affects the behaviors of ESCs, we conducted the first mechanomics analysis of hESCs under fluid shear. Functional tests under shear flow exemplified the interplay between nucleus state and mechanical loading and, accordingly, a novel working model was brought into light. Specifically, CFL2, one type of F-actin binding proteins (Fig. 7a), was upregulated under fluid shear, which caused F-actin network reorganization especially at the nuclear periphery. This action subsequently increased nucleus spreading, and the alteration of nuclear shape then enhanced H2B acetylation and induced global chromatin decondensation (Fig. 7b). These epigenetic alterations thereby promoted rRNA synthesis, softened the nucleus, and correlated positively with cell-cell contact (Fig. 7c). More importantly, applying fluid shear could modify the primed state of hESCs upon the above mechanism (Fig. 7d). Thus, this work provided a novel insight into how fluid shear signals trigger biochemical responses in the nucleus and highlighted the key role of mechanoepigenetics in stem cell mechanobiology.

Mechanical forces are able to induce histone acetylation on various types of cells in cellular mechanoepigenetic responses to mechanical/physical microenvironment. For instance, mechanical loading and surface micropatterning modulate H3 acetylation level and nuclear shape of MSCs [18]. In three-dimensional as well as non-adhesive or micropatterned cultures, human mammary epithelial cells tend to be rounded up and undergo deacetylation of histones H3 and H4 independent of biochemical cues [54]. Complemented with these cues, we demonstrated, for the first time, that long-term application of fluid shear enhanced H2B acetylation when TSA, which inhibits histone deacetylase activity and leads to acetylation restoration, serves as a chemical contrast. Increased acetylation can be derived from both the acetylation restoration by HDAC inhibitors and histone acetylatransferase (HAT)-mediated acetylation [55, 56]. In the current work, TSA-induced H2B acetylation level was lower than that under shear flow, implying that fluid shear may increase HAT recruitment to chromatin. CFL2 accumulation at nuclear periphery (Fig. 3j and Additional file 4: Table S1) indicated an active F-actin dynamics. Since F-actin is able to bind HAT to mediate chromatin remodeling [57, 58] and cofilin family proteins have the ability to chaperon the actins to the nucleus [59, 60], the chromatin could strongly interact with HAT via the altered F-actin network in the nucleus. As for transmitting external forces to F-actin, it is likely achieved by alterations of focal adhesion since cell adhesion is mainly mediated by focal adhesion complexes [61] and focal adhesion gene set was enriched (Additional file 2: Figure S2f). Here hESCs under fluid shear were ready to digest with trypsin on downregulated ITGB1 (Additional file 7: Figure S6), suggesting that focal adhesion is impaired by fluid shear. Hereto, a canonical mechanotransmission pathway, including adhesion molecule, cytoskeleton, nuclear matrix, and chromatin, was delineated.

Nuclear mechanics is also regulated by external forces via cytoskeletal reorganization and nuclear remodeling, mainly upon the above mechanotransmission pathway. On one hand, the cytoskeleton could transmit external force to mechanosensitive structures within a cell and initiate mechanical remodeling of the nucleus via cytoskeletal network [37]. In a recent work, a theoretical model of mechanical homeostasis of the nucleus was proposed in close relation to F-actin network reorganization [38]. In the current work, the GSEA analysis proposed the enrichment of “nuclear matrix” (Additional file 2: Figure S2d) and “nuclear periphery” (Additional file 2: Figure S2e), all of which are linked to cytoskeleton [44]. Lamin matrix proteins could regulate nuclear stiffness [20], and chromatin decondensation also induces nuclear softening in ESCs [16]. Thus, not only lamin proteins but also chromatin architectures contribute to nuclear mechanics [62]. Along this line, the enlarged, softened nucleus was observed here under shear flow, which can be seen as the biomechanical responses at subcellular level and implies the mechanical adoption to implement new biological functions.

Mechanotransductive responses presented here have potential biological implications. ESCs can proliferate indefinitely in vitro without apparent contact inhibition [63], but no canonical explanation is presented for the loss of contact inhibition. hESCs cultured in feeder-free conditions experience an epithelial-mesenchymal transition (EMT), with mesenchymal-like cells presenting at the periphery but epithelium-like cells inside the colonies [64]. hESCs at the periphery had higher AcH2B/DNA ratio than that in the inner (Fig. 4a), implying a possible correlation of H2B acetylation with EMT. Indeed, H2B acetylation could regulate EMT in trophoblast stem cells, which is accompanied by the loss of contact inhibition [65]. Thus, a link between H2B acetylation and contact inhibition could be established to further understand the related issues in hESC renewal. ESCs have a higher acetylation and less compact euchromatin than their differentiated counterpart [66], supporting the understandings on the loss of contact inhibition of hESCs.

Although ESCs are usually classified into naive and primed states, recent studies sound a note that these states represent a continuum of configurations and various degrees of naivety or priming exist between them [67], or so-called hierarchy of pluripotency. Among these states, distinct chromatin architecture has different physiological significances, which affects nuclear mechanics and is regulated by mechanical forces [16, 17, 20]. In an integrated model of forces on chromatin, chromatin can regulate transcription by its condensation state and global architecture under external forces [59]. Indeed, the open state of chromatin facilitates the transcription of *DHFR* gene under forces [68], while force-induced chromatin compaction results in polycomb-mediated promoter silencing during differentiation [69]. Upon these bases, we proposed here that the primed state of hESCs under fluid shear yields higher level of acetylation and less compacted chromatin. These features confer hESCs more capacity to control gene expression through mechanosensitive transcription regulation. Namely, shear stress enlarges the volume of nuclei and decondenses chromatin, providing more space available in the nucleus to facilitate the entry of differentiation-inducing factors and transcription factors to initiate transcription [16]. This can be partly proven by the increased rRNA synthesis in the current work (Fig. 6e), since the upregulation of rRNA means that hESCs recruit more transcription factors necessary for rDNA transcription. While stem cells are always committed to lineage specification before mechanical loading or mechanical loading and differentiation inducers are usually coupled [9, 10], hESCs were exposed to fluid shear alone in this work, thereby decoupling their interplay with biochemical factors and isolating the responses of hESCs to fluid shear but not their differentiating counterparts. Our observations suggest that hESCs under shear flow maintain pluripotency and likely make differentiation-inducing factors more efficient by opening the target region of chromatin and thus facilitates the lineage specification. And it is not surprising that fluid shear can also facilitate lineage specification in the presence of differentiation media with specific differentiation-inducing factors, since both biochemical and biomechanical cues are coupled together in vivo.

Recent studies also demonstrate mechanical regulation of nuclear deformation and chromatin state of stem cells [70, 71]. While those works mainly focus on the differentiation process (from stem cells to lineage-specific cells), the current work aimed to elucidate the upstream events (different configurations of the primed state in stem cells). Despite the biological significances mentioned above, it seems that mechanical cues are not specific to stem cell differentiation but more likely play a neutral role among various cell types, e.g., to induce epigenetic modification and increase reprogramming efficiency in fibroblast [19, 72]. Thus, it is arguably proposed that mechanical cues are utilized to prepare the cells for biochemical signals to take effect through altering chromatin architecture, a process that depends on various loading parameters. In this work, only one set of steady flow parameters with a shear stress of 1.1 Pa for 24 h was applied in the related functional studies, partially because this set can mimic the blood flow acting on hepatic sinusoids to modulate liver regeneration [26, 73]. Evidently, the impacts of entire sets of fluid flow parameters on hESC state are worthwhile of future studies.

## Conclusions

In summary, mechanomics analysis and function study for hESCs under fluid shear demonstrated that shear stress alters nuclei morphology via F-actin/CFL2 cytoskeleton reorganization, resulting in enhanced H2B acetylation and chromatin decondensation as well as reduction in nucleus stiffness. These responses make hESCs be more prone to adopt transcription factors for consolidating the primed state.

## Supplementary information

**Additional file 1: Figure S1.** Schematic of customized flow chamber system. hESCs were placed onto the substrate of parallel-plate chamber and then exposed to shear flow at given flow parameters in Fig. 1A.

**Additional file 2: Figure S2.** GSEA analysis was performed to identify enrichment of gene sets (see Materials and methods). The bar-code plot indicates the position of pre-ranked proteins with *red* and *blue* colors marking gene up-regulation and down-regulation, respectively. FDR, false discovery rate.

**Additional file 3: Figure S3.** Pairwise matrix analysis. Pairwise plot matrix was created to conduct the pairwise comparisons of nucleus area, DNA, AcH2B and AcH2B/DNA under steady shear of 1.1 Pa for 24 h, where the density and correlation coefficient of the respective variables were displayed along the diagonal or on the upper triangle. *Black points* denote individual colonies measured, and *gray shade* indicates 95% confidence interval of linear fitting.

**Additional file 4: Table S1.** Core enrichment of proteins related to nuclear matrix and nuclear periphery identified using iTRAQ.

**Additional file 5: Figure S4.** Actin expression of hESCs under steady shear of 1.1 Pa for 24 h. Mean F-actin intensity under static control or fluid shear, as well as normalized mean F-actin intensity under fluid shear (= mean F-actin intensity multiplied by cell area under fluid shear and divided by cell area under static control) were illustrated. The numbers of tested replicates and measured colonies, (M, m), are (3, 24) for static control and (3, 45) for fluid shear.

**Additional file 6: Figure S5.** NANOG expression of hESCs under steady shear of 1.1 Pa for 24 h. Isotype control (*red*) or NANOG (*cyan*) fluorescence intensity was detected using flow cytometry analysis.

**Additional file 7: Figure S6.** Trypsin digestion and ITGB1 expression. Optical images of static or sheared hESCs were shown in *A* and the quantified ITGB1 protein expression was present in *B*. *Red circles* in *A* indicated the cells being detached from the substrate. Flow direction was indicated by *black arrow*. Bar = 100 μm in *A*. *S*, steady flow; *P*, pulsatile flow; 0.5, 0.5 Pa; 1.1, 1.1 Pa.

## Data Availability

The datasets generated during and/or analyzed during the current study are available from the corresponding author on reasonable request.

## Abbreviations

AFM: Atomic force microscopy
EMT: Epithelial-mesenchymal transition
ESCs: Embryonic stem cells
FC: Fold change
FCM: Flow cytometry
FDR: False discovery rate
GSEA: Gene set enrichment analysis
HAT: Histone acetylatransferase
HDAC: Histone deacetylase
ICQ: Intensity correlation analysis
IF: Immunofluorescence
iTRAQ: Relative and absolute quantitation
MS: Mass spectrometry
MSC: Mesenchymal stem cells
qPCR: Quantitative real-time polymerase chain reaction
ROIs: Regions of interest
SCX: Strong cation exchange
TSA: Trichostatin A
WB: Western blotting
