# Supplementary figures and images for: Flow-enhanced priming of hESCs through H2B acetylation and chromatin decondensation
Source: Stem Cell Res Ther. 2019 Nov 27;10:349. doi: 10.1186/s13287-019-1454-z (PMC6880446; doi:10.1186/s13287-019-1454-z)

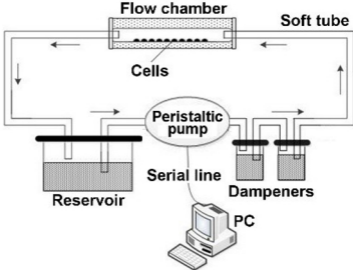

Supplement: Supplementary file 1 — Additional file 1: Figure S1. Schematic of customized flow chamber system. hESCs were placed onto the substrate of parallel-plate chamber and then exposed to shear flow at given flow parameters in Fig. 1A. [file 13287_2019_1454_MOESM1_ESM.pdf]

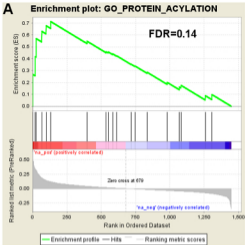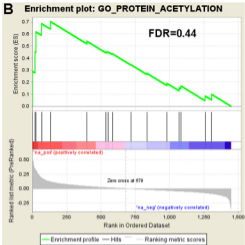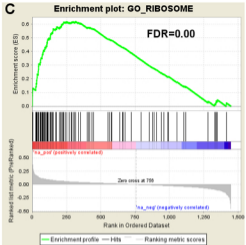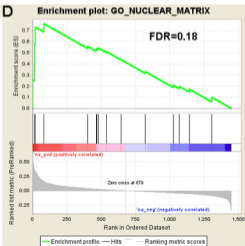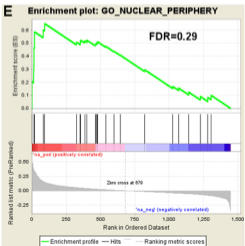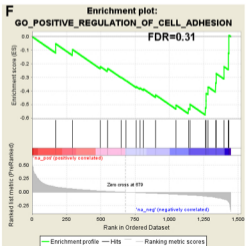

Supplement: Supplementary file 2 — Additional file 2: Figure S2. GSEA analysis was performed to identify enrichment of gene sets (see Materials and methods). The bar-code plot indicates the position of pre-ranked proteins with red and blue colors marking gene up-regulation and down-regulation, respectively. FDR, false discovery rate. [file 13287_2019_1454_MOESM2_ESM.pdf]

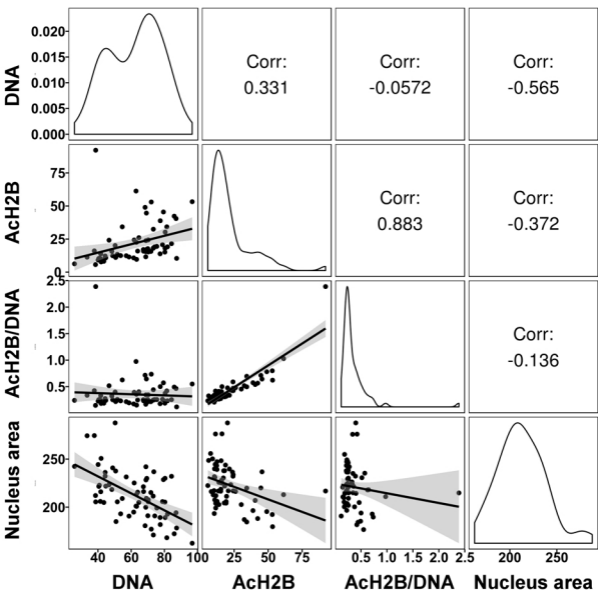

Supplement: Supplementary file 3 — Additional file 3: Figure S3. Pairwise matrix analysis. Pairwise plot matrix was created to conduct the pairwise comparisons of nucleus area, DNA, AcH2B and AcH2B/DNA under steady shear of 1.1 Pa for 24 h, where the density and correlation coefficient of the respective variables were displayed along the diagonal or on the upper triangle. Black points denote individual colonies measured, and gray shade indicates 95% confidence interval of linear fitting. [file 13287_2019_1454_MOESM3_ESM.pdf]

Mean fluorescence intensity (A.U.)

160

120

80

40

$P=3.81 \times 10^{-3}$

Static

Shear

Normalized

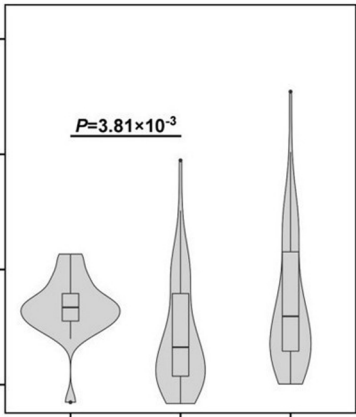

Supplement: Supplementary file 5 — Additional file 5: Figure S4. Actin expression of hESCs under steady shear of 1.1 Pa for 24 h. Mean F-actin intensity under static control or fluid shear, as well as normalized mean F-actin intensity under fluid shear (= mean F-actin intensity multiplied by cell area under fluid shear and divided by cell area under static control) were illustrated. The numbers of tested replicates and measured colonies, (M, m), are (3, 24) for static control and (3, 45) for fluid shear. [file 13287_2019_1454_MOESM5_ESM.pdf]

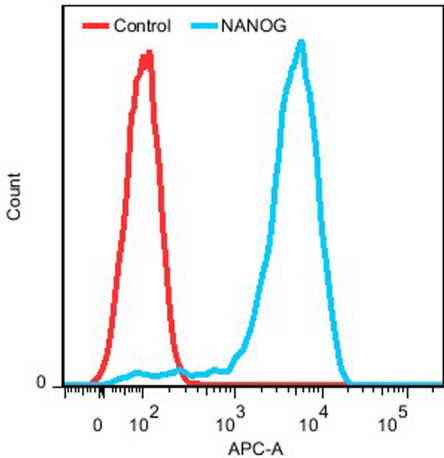

Supplement: Supplementary file 6 — Additional file 6: Figure S5. NANOG expression of hESCs under steady shear of 1.1 Pa for 24 h. Isotype control (red) or NANOG (cyan) fluorescence intensity was detected using flow cytometry analysis. [file 13287_2019_1454_MOESM6_ESM.pdf]

**A****Static**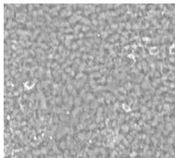**Shear**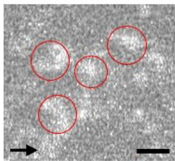**B**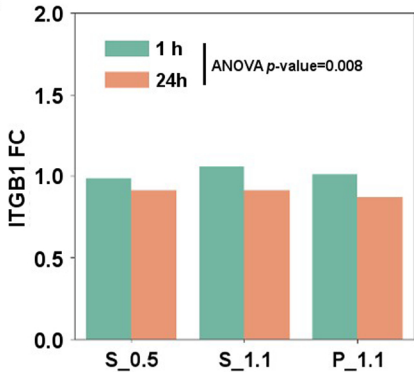

Supplement: Supplementary file 7 — Additional file 7: Figure S6. Trypsin digestion and ITGB1 expression. Optical images of static or sheared hESCs were shown in A and the quantified ITGB1 protein expression was present in B. Red circles in A indicated the cells being detached from the substrate. Flow direction was indicated by black arrow. Bar = 100 μm in A. S, steady flow; P, pulsatile flow; 0.5, 0.5 Pa; 1.1, 1.1 Pa. [file 13287_2019_1454_MOESM7_ESM.pdf]
