# Supplementary material for: Flow-enhanced priming of hESCs through H2B acetylation and chromatin decondensation
Source: Stem Cell Res Ther. 2019 Nov 27;10:349. doi: 10.1186/s13287-019-1454-z (PMC6880446; doi:10.1186/s13287-019-1454-z)
Supplement: Supplementary file 4 — Additional file 4: Table S1. Core enrichment of proteins related to nuclear matrix and nuclear periphery identified using iTRAQ. [file 13287_2019_1454_MOESM4_ESM.docx]

| Protein | Annotation | FC | *p*-value | FDR |
| --- | --- | --- | --- | --- |
| PPIG | Cyclophilin G | 1.55 | 0.01 | 0.18 |
| CENPF | Centromere protein F | 1.36 | 0.10 | 0.26 |
| ZNF326 | Zinc finger protein 326 | 1.36 | 0.04 | 0.23 |
| CFL2 | Cofilin2 | 1.34 | 0.01 | 0.18 |
| AKAP8 | A-Kinase Anchoring Protein 8 | 1.16 | 0.03 | 0.23 |
